# Constriction of juxta-ductal aorta and rapid progression of obstruction in a newborn

**DOI:** 10.4103/0974-2069.74054

**Published:** 2010

**Authors:** Neeraj Awasthy, Munesh Tomar, Sitaraman Radhakrishnan, Krishna Subramoney Iyer

**Affiliations:** Department of Pediatric and Congenital Heart Diseases, Escorts Heart Institute and Research Centre, New Delhi, India

**Keywords:** Aortic coarctation, arterial duct, echocardiography

## Abstract

A 13-day-old baby girl presenting with features of congestive cardiac failure was found to have coarctation of the aorta (CoA) and patent ductus arteriosus (PDA) by echocardiography. Doppler spectral display revealed moderate CoA. Echocardiogram, 12 hours later, showed severe juxtaductal aortic coarctation with spontaneous closure of PDA. This case emphasises the need to keep a close watch on the progress of CoA in the neonatal period, even if the duct has narrowed to a small size thus demonstrating the role of constriction of juxtaductal aorta in pathogenesis of coaractation. Closure of even asmall PDA can cause acute progression CoA in the presence of posterior shelf.

## INTRODUCTION

Coarctation of aorta (CoA), which occurs in l/l2 000 live births and accounts for 7.5% of all congenital heart defects,[1] may present in the neonatal periods with signs of severe heart failure and vascular collapse with constriction of the ductus arteriosus. We report a newborn with acute onset severe aortic coarctation following closure of a small patent ductus arteriosus (PDA). The case emphasises the central role of the ductal tissue in pathogenesis of the coarctation. The clinical implications are discussed.

## CASE REPORT

A 13-day-old baby girl was born full term with a birth weight of 2600 g. The treating neonatologist suspected a congenital heart disease and the child was referred to our center for further workup. On her arrival, the child had features of congestive cardiac failure (tachycardia, tachypnea, feeding difficulty). Chest roentgenogram revealed mild cardiomegaly (cardiothoracic ratio 0.65) with pulmonary venous congestion. Electrocardiogram showed sinus tachycardia with right ventricular hypertrophy and right axis deviation. Echocardiography disclosed a fossa ovalis ASD (6mm) with left to right shunt, bicuspid aortic valve, with laminar flow, a small PDA shunting left to right, mild juxta ductal CoA with posterior shelf [Figure 1a], mild tricuspid regurgitation (TR) (TR peak gradient – 70 mm Hg) with mild biventricular dysfunction. Doppler interrogation at the site of coarctation revealed a peak systolic gradient of 30 mmHg, with no diastolic spilling [Figure 1b]. In view of respiratory distress, the baby was started on intravenous ionotropes (low dose Dopamine and Dobutamine), intravenous diuretics (Frusemide) and was put on positive pressure ventilation with adequate analgesia. Arterial blood gas analysis showed no significant acidosis, though lactate level was high (4mmol/L). After 12 hours, echocardiography was reviewed and showed severely narrowed coarctation at juxta ductal portion with a peak systolic gradient of 64 mmHg with diastolic spilling, poor left ventricular contractility (left ventricular ejection fraction: 45%) and closed ductus arteriosus [Figures 2a and b]. Surgery confirmed the echocardiographic diagnosis of juxta ductal CoA. She underwent successful aortoplasty with end-to-end anastomosis with ductus division and was discharged uneventfully two week later.

**Figure 1a:**
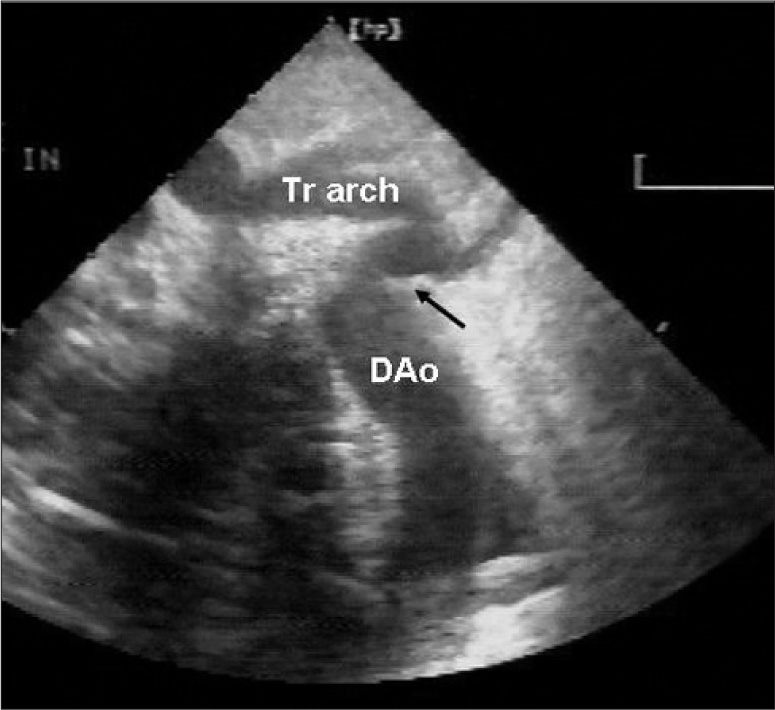
Suprasternal long axis view of the aortic arch showing a posterior shelf (arrow)

**Figure 1b:**
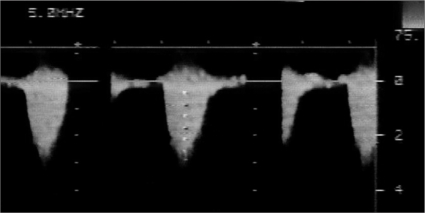
Continuous wave Doppler interrogation across the arch before closure of duct, showing peak systolic gradient of 30 mm Hg, with no diastolic spill

**Figure 2a:**
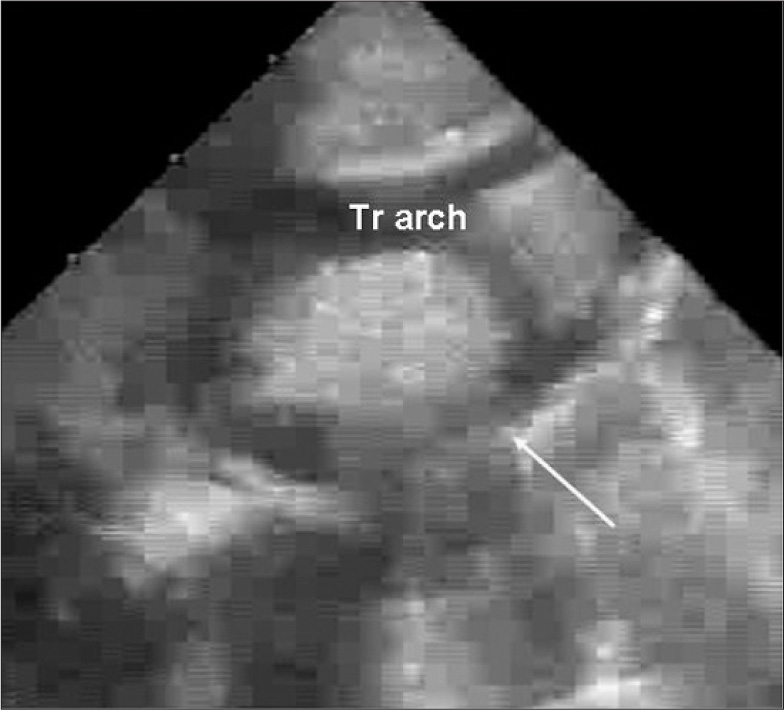
Suprasternal long axis view of the aortic arch performed 12 hours later showed the juxtaductal segment was severely narrowed (arrow) after ductal closure

**Figure 2b:**
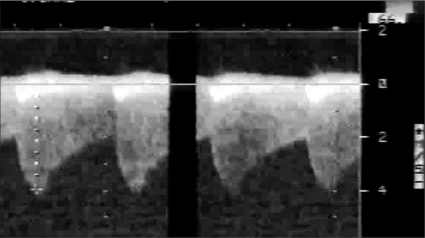
Continuous wave Doppler interrogation across the arch from suprasternal view after ductal closure showing systolic gradient 64 mm Hg, with diastolic spilling (continuous flow). Tr arch-transverse arch, Dao-descending aorta

## DISCUSSION

Coarctation of the aorta is commonly associated with PDA. The latter is not only implicated in the morphogenesis of CoA (juxtaductal presence of ductal tissue), but also is known to have an impact on its hemodynamics particularly in neonates. It is well recognized that in the majority of these very young patients the aortic flow, particularly in the descending aorta, may be dependent on the patency of the ductus arteriosus. This was supported in clinical practice by the sudden rapid deterioration of the infant’s condition following closure of the ductus arteriosus. Thus the term ‘ductal dependent’ coarctation or circulation was coined. These phenomena were seen more often with the discovery of prostaglandin to reopen the closed ductus arteriosus resulting in rapid improvement of the patient’s cardiovascular status by re-establishing descending aortic blood flow.[2–4] On the other hand is the theory that proposes the presence of the ductal tissue in the aortic wall responsible for the pathogenesis of CoA. It is hypothesized that ductal tissue may extend out to the aortic isthmus in the case of CoA. In studies conducted by Ho and Anderson,[5] a sling of ductal tissue has been found around the aortic isthmal orifice in 100% of their patients with coarctation or tubular hypoplasia of the aortic arch It is this close relationship with the ductus arteriosus that is perhaps of paramount importance when it comes to managing very young patients with coarctation.[1]

Our case highlights the fact that spontaneous closure of even a small PDA caused rapid progression of CoA further strengthening the role of ductal tissue pathogenesis of coarctation. It is unlikely that the ductus itself maintained the flow into the descending aorta because it was a small PDA at the time of the initial examination. What is more likely is that closure of the ductus further tightened the “noose” around an already preexisting coarctation shelf. This substrate can be recognized on echocardiography if one sees a posterior aortic shelf on the high parasternal “ductal view” or suprasternal view and should alert the physician for close follow-up of these neonates. Hence it is important to observe all newborns with a small isthmus and posterior shelf for development of CoA and should be discharged only after ensuring that there is no obstruction after the PDA has completely closed.
